# COVID‐19 outcomes in patients with cancer: Findings from the University of California health system database

**DOI:** 10.1002/cam4.4604

**Published:** 2022-03-09

**Authors:** Daniel H. Kwon, Jose Cadena, Sam Nguyen, Kwan Ho Ryan Chan, Braden Soper, Amy L. Gryshuk, Julian C. Hong, Priyadip Ray, Franklin W. Huang

**Affiliations:** ^1^ Division of Hematology/Oncology, Department of Medicine University of California San Francisco California USA; ^2^ Computational Engineering, Engineering Directorate Lawrence Livermore National Laboratory California USA; ^3^ Center for Applied Scientific Computing, Computing Directorate Lawrence Livermore National Laboratory California USA; ^4^ Physical and Life Sciences Directorate Lawrence Livermore National Laboratory California USA; ^5^ Helen Diller Family Comprehensive Cancer Center University of California San Francisco San Francisco California USA; ^6^ Bakar Computational Health Sciences Institute University of California San Francisco California USA; ^7^ Department of Radiation Oncology University of California San Francisco California USA; ^8^ Department of Electrical and Computer Engineering Johns Hopkins University Baltimore Maryland USA

**Keywords:** cancer, COVID‐19, myeloproliferative neoplasm, outcomes research

## Abstract

**Background:**

The interaction between cancer diagnoses and COVID‐19 infection and outcomes is unclear. We leveraged a state‐wide, multi‐institutional database to assess cancer‐related risk factors for poor COVID‐19 outcomes.

**Methods:**

We conducted a retrospective cohort study using the University of California Health COVID Research Dataset, which includes electronic health data of patients tested for severe acute respiratory syndrome coronavirus 2 (SARS‐CoV‐2) at 17 California medical centers. We identified adults tested for SARS‐CoV‐2 from 2/1/2020–12/31/2020 and selected a cohort of patients with cancer. We obtained demographic, clinical, cancer type, and antineoplastic therapy data. The primary outcome was hospitalization within 30d after the first positive SARS‐CoV‐2 test. Secondary outcomes were SARS‐CoV‐2 positivity and severe COVID‐19 (intensive care, mechanical ventilation, or death within 30d after the first positive test). We used multivariable logistic regression to identify cancer‐related factors associated with outcomes.

**Results:**

We identified 409,462 patients undergoing SARS‐CoV‐2 testing. Of 49,918 patients with cancer, 1781 (3.6%) tested positive. Patients with cancer were less likely to test positive (RR 0.70, 95% CI: 0.67–0.74, *p* < 0.001). Among the 1781 SARS‐CoV‐2‐positive patients with cancer, BCR/ABL‐negative myeloproliferative neoplasms (RR 2.15, 95% CI: 1.25–3.41, *p* = 0.007), venetoclax (RR 2.96, 95% CI: 1.14–5.66, *p* = 0.028), and methotrexate (RR 2.72, 95% CI: 1.10–5.19, *p* = 0.032) were associated with greater hospitalization risk. Cancer and therapy types were not associated with severe COVID‐19.

**Conclusions:**

In this large, diverse cohort, cancer was associated with a decreased risk of SARS‐CoV‐2 positivity. Patients with BCR/ABL‐negative myeloproliferative neoplasm or receiving methotrexate or venetoclax may be at increased risk of hospitalization following SARS‐CoV‐2 infection. Mechanistic and comparative studies are needed to validate findings.

## Graphical abstract

In a large dataset of patients who underwent SARS‐CoV‐2 testing across 17 California medical centers, we conducted multivariable analyses to evaluate cancer‐related risk factors for poor COVID‐19 outcomes. We found that patients with cancer had a lower risk of SARS‐CoV‐2 positivity than those without cancer. BCR/ABL‐negative myeloproliferative neoplasms, venetoclax use, and methotrexate use were associated with an increased risk of hospitalization after a COVID‐19 diagnosis.

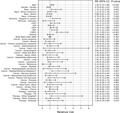

## 1 INTRODUCTION

The severe acute respiratory syndrome coronavirus 2 (SARS‐CoV‐2) pandemic has caused over 5.5 million deaths worldwide.^1^ The relationship between cancer and SARS‐CoV‐2 infections and outcomes remains a topic of ongoing controversy, and of importance to both oncologists and their patients.

Thus far, most studies investigating these relationships have been limited in size or detail. Though several studies have identified associations between SARS‐CoV‐2 infection or outcomes and some cancer diagnoses, such as lung and hematologic malignancies,^2^, ^3^, ^4^, ^5^, ^6^, ^7^, ^8^, ^9^, ^10^ large cohort studies of patients with cancer and COVID‐19 have grouped malignancies or excluded certain cancer types, such as myeloproliferative neoplasms.^4^, ^7^, ^10^ Since there is heterogeneity in the biology and treatment of cancer based on specific cancer histologies, outcomes following SARS‐CoV‐2 infection may be differentially impacted by cancer type. Deeper investigation of outcomes based on cancer type is needed.

Moreover, it remains uncertain whether recent antineoplastic systemic therapy use, particularly cytotoxic chemotherapy, is a risk factor for poor COVID‐19 outcomes. A recent meta‐analysis of 26 cohort studies found no adverse effect of various anti‐cancer therapies on COVID‐19 severity or mortality,^11^ perhaps due to the categorization of heterogeneous groups of therapies. Detailed examination of outcomes based on specific therapies is needed. Clarifying these cancer‐related risk factors is useful in counseling and management of patients with cancer and COVID‐19.

Informatics‐based analyses of large, real‐world data sets may facilitate an understanding of the relationships between these potential risk factors and COVID‐19 outcomes. We leveraged the University of California Health COVID Research Data Set (UC CORDS),^12^ which aggregates the electronic health records data of all patients who underwent testing for SARS‐CoV‐2 at University of California (UC)‐affiliated hospitals. We hypothesized that understudied cancer types, for example, hematologic cancer subtypes and specific systemic therapies, such as lymphocyte‐depleting therapies, are associated with higher hospitalization, intensive care use, and death following COVID‐19.

## 2 METHODS

We conducted a retrospective cohort study of patients using UC CORDS v2.0.^12^ This limited data set includes prospectively‐collected electronic health data of all patients who underwent quantitative reverse transcription‐polymerase chain reaction (RT‐qPCR) testing for SARS‐CoV‐2 at 5 UC academic medical centers (Davis, Irvine, Los Angeles, San Diego, and San Francisco) and 12 affiliated California hospitals. UC CORDS is organized using the Observational Medical Outcome Partnership common data model, which contains diagnoses, medications, labs, and procedures associated with clinical encounters. Data are refreshed on a weekly basis. The study protocol was reviewed and approved by both UCSF and Lawrence Livermore National Laboratory institutional review boards.

### 2.1 Cohort definitions

We identified patients who underwent a SARS‐CoV‐2 RT‐qPCR test between February 1, 2020, and December 31, 2020, and were ≥ 18‐years‐old at the first test date to create the first cohort. We then identified a second cohort of patients with cancer, defined as ≥1 clinical encounter associated with a cancer ICD‐10‐CM code (C*, D45, D46, and D47* excluding monoclonal gammopathy D47.2) within 1 year prior to the test date, that is, “index date.” For patients with a positive SARS‐CoV‐2 RT‐qPCR test, the index date was the first positive date; otherwise, the index date was the first negative test date. These criteria were intended to select patients being routinely followed for their cancer. Patients with only basal and squamous cell cutaneous cancers were excluded given the extremely low morbidity and mortality of these cancers. Patients with other/unknown gender were excluded, since none of these patients had both cancer and COVID‐19. For analysis of severe COVID‐19 (defined below), a third cohort of cancer patients with COVID‐19 who were hospitalized within 30 days of index date was created to enrich for laboratory data and likelihood that outcomes are attributable to COVID‐19. Figure S1 shows a flow diagram of these three cohorts.

### 2.2 Independent variables

For demographic variables, we included birth year, gender, race, and ethnicity. For clinical variables, we included cancer type; comorbidities that are known to be associated with COVID‐19 severity in patients with cancer (coronary artery disease, congestive heart failure, chronic kidney disease, chronic obstructive pulmonary disease, and asthma within 1 year prior to index date); and body mass index.^13^ Primary cancer types were granularly categorized as hematologic (acute leukemia, chronic lymphocytic leukemia/small lymphocytic lymphoma, chronic myeloid leukemia, lymphoma, myelodysplastic syndrome, BCR/ABL‐negative myeloproliferative neoplasm, plasma cell dyscrasia, and other); solid (breast, gastrointestinal, germ cell, gynecological, head and neck, hepatobiliary/pancreatic, lung, melanoma, nervous system, neuroendocrine/endocrine, prostate, sarcoma, urinary tract, and other); multiple cancer types; and unspecified cancer type (Table S1). Antineoplastic systemic therapy use from 60 days prior to index date to 30 days after was included. Antineoplastic systemic therapies were categorized by adapting the National Library of Medicine RxClass into antibody, chemotherapy, hormone therapy, immune‐based therapy (includes agents that stimulate or modulate the immune system), tyrosine kinase inhibitor, other cytotoxic therapy, and other targeted therapy (Table S2).^14^ We also included laboratory data from 60 days prior to the index date to 30 days afterward.

### 2.3 Dependent variables

Outcomes included SARS‐CoV‐2 positivity (at least 1 positive RT‐qPCR test); hospitalization within 30 days after the index date; and a composite endpoint for severe COVID‐19, defined as either intensive care unit admission, need for mechanical ventilation, or death within 30 days after the index date.

### 2.4 Statistical analysis

We first calculated the incidence of SARS‐CoV‐2 test positivity among all patients tested for SARS‐CoV‐2, regardless of cancer. Then, we conducted a series of multivariable logistic regression models for each of the three cohorts described above (all COVID‐19‐positive patients, COVID‐19‐positive patients with cancer, and hospitalized COVID‐19‐positive with cancer). To predict the risk of SARS‐CoV‐2 test positivity in the overall cohort, we created a multivariable model that included age, gender, race, ethnicity, comorbidities, any cancer history, and receipt of any systemic therapy. Laboratory tests were not included given the high rate of missing values. To identify cancer‐related risk factors for hospitalization in the cohort of patients with cancer and COVID‐19 (the primary outcome), we created two multivariable models: one in which systemic therapies were categorized and another in which individual therapies were delineated. Both models contained specific cancer types compared to unspecified cancer type, selected since this category comprised of non‐specific diagnostic codes that would likely span a range of cancer types (e.g., ICD‐10 C79.51 secondary neoplasm of bone). Again, laboratory tests were not included in these models given the high rate of missing values. Lastly, to evaluate the risk of severe COVID‐19, we used the cohort restricted to hospitalized patients with cancer and COVID‐19. We created a multivariable model with systemic therapies as categories and laboratory tests as continuous variables, only including tests that at least 70% of patients had completed. We did not incorporate individual therapies in this analysis because few patients were associated with each therapy.

Logistic regressions were visualized using forest plots with adjusted relative risk ratios, and 95% confidence intervals (*p* < 0.05) were considered significant. Multiple imputations were used for imputation of missing laboratory values.^15^ We did not correct for multiple comparisons given the exploratory nature of the study.^16^ The logistic regression models were implemented using the statsmodels module in the Python programming language (v3.8).^17^

## 3 RESULTS

Overall, 24,177 of 409,462 (5.9%) patients undergoing SARS‐CoV‐2 RT‐qPCR tested positive for SARS‐CoV‐2 at any time during the study period. Of the 49,918 patients with a history of cancer, 1781 (3.6%) tested positive. The mean age of SARS‐CoV‐2‐positive patients with cancer was 59 years (SD = 16); 950 (53%) were female, 939 (53%) were White, and 636 (36%) were Hispanic or Latino (Table 1). The most common cancer types were Multiple (*N* = 293, 16%); Breast (*N* = 241, 14%); and Prostate (*N* = 122, 7%) per Table 1. Three hundred twenty‐four (18%) patients were on active systemic therapy, of which chemotherapy was the most common (N = 153, 9%). Individual therapies are listed in Table S3.

**TABLE 1:** Characteristics of patients undergoing SARS‐CoV‐2 testing

Variables	All patients (N = 409,462) No. (%)	Patients with cancer (N = 49,918) No. (%)	Patients with cancer and COVID‐19 (N = 1781) No. (%)
*Age*			
18–65	294,375 (71.89)	24,625 (49.33)	1044 (58.62)
65–75	68,413 (16.71)	14,383 (28.81)	420 (23.58)
>=75	46,674 (11.40)	10,910 (21.86)	317 (17.80)
*Gender*			
Female	227,493 (55.56)	25,667 (51.42)	950 (53.34)
Male	181,969 (44.44)	24,251 (48.58)	831 (46.66)
*Race*			
Asian	39,882 (9.74)	5296 (10.61)	152 (8.54)
Black or African‐American	22,164 (5.41)	2407 (4.82)	108 (6.06)
White	222,022 (54.22)	32,698 (65.51)	939 (52.72)
Other	40,830 (9.97)	4624 (9.26)	278 (15.61)
Unknown	84,564 (20.66)	4893 (9.80)	304 (17.07)
*Ethnicity*			
Hispanic or Latino	67,046 (16.37)	7596 (15.21)	636 (35.71)
Not Hispanic or Latino	287,647 (70.25)	40,780 (81.70)	1104 (61.99)
Unknown	54,769 (13.38)	1542 (3.09)	41 (2.30)
*Comorbidities*			
Coronary artery disease	9427 (2.30)	2090 (4.19)	129 (7.24)
Congestive heart failure	16,348 (3.99)	3476 (6.96)	192 (10.78)
Chronic kidney disease	21,919 (5.35)	5522 (11.06)	273 (15.33)
Diabetes mellitus	37,553 (9.17)	7681 (15.38)	474 (26.61)
Chronic obstructive pulmonary disease	11,693 (2.86)	3275 (6.56)	132 (7.41)
Asthma	22,686 (5.54)	3449 (6.91)	162 (9.10)
*Body mass index, kg/m* ^*2*^			
<18.5	10,092 (2.47)	1975 (3.96)	61 (3.43)
18.5–25	98,938 (24.16)	15,620 (31.29)	472 (26.50)
25–30	89,680 (21.90)	14,330 (28.71)	532 (29.87)
≥30	72,768 (17.77)	10,698 (21.43)	481 (27.01)
Missing	137,984 (33.70)	7295 (14.61)	235 (13.19)
*Cancer types*			
Hematologic cancer			
Acute leukemia	570 (0.14)	570 (1.14)	41 (2.30)
Chronic lymphocytic leukemia/small lymphocytic lymphoma	516 (0.13)	516 (1.03)	23 (1.29)
Chronic myeloid leukemia	167 (0.04)	167 (0.33)	11 (0.62)
Lymphoma	1690 (0.41)	1690 (3.39)	76 (4.27)
Myelodysplastic syndrome	234 (0.06)	234 (0.47)	10 (0.56)
Myeloproliferative neoplasm	1432 (0.35)	1432 (2.87)	97 (5.45)
Plasma cell dyscrasia	1114 (0.27)	1114 (2.23)	54 (3.03)
Hematologic, Other	214 (0.05)	214 (0.43)	9 (0.51)
Solid cancer			
Breast	6273 (1.53)	6273 (12.57)	241 (13.53)
Gastrointestinal	3467 (0.85)	3467 (6.95)	96 (5.39)
Germ cell	230 (0.06)	230 (0.46)	5 (0.28)
Gynecological	2996 (0.73)	2996 (6.00)	119 (6.68)
Head and neck	2047 (0.50)	2047 (4.10)	46 (2.58)
Hepatobiliary/pancreatic	2346 (0.57)	2346 (4.70)	83 (4.66)
Lung	1404 (0.34)	1404 (2.81)	33 (1.85)
Melanoma	1564 (0.38)	1564 (3.13)	32 (1.80)
Nervous system	488 (0.12)	488 (0.98)	14 (0.79)
Neuroendocrine/endocrine	2435 (0.59)	2435 (4.88)	76 (4.27)
Prostate	4653 (1.14)	4653 (9.32)	122 (6.85)
Sarcoma	721 (0.18)	721 (1.45)	17 (0.95)
Urinary tract	2028 (0.49)	2028 (4.06)	68 (3.82)
Solid tumor, other	2200 (0.54)	2200 (4.40)	74 (4.15)
Multiple cancers	6730 (1.64)	6730 (13.48)	293 (16.45)
Unspecified cancer type	4399 (1.07)	4399 (8.81)	141 (7.92)
*Cancer systemic therapies*			
Antibody therapy	1329 (0.32)	1126 (2.26)	41 (2.32)
Chemotherapy	6331 (1.55)	3966 (7.94)	153 (8.59)
Hormonal therapy	3762 (0.92)	2742 (5.49)	86 (4.83)
Immune‐based therapy	62 (0.02)	55 (0.11)	0 (0.00)
Other targeted therapy	452 (0.11)	420 (0.84)	18 (1.01)
Other cytotoxic therapy	1417 (0.35)	858 (1.72)	39 (2.19)
Tyrosine kinase inhibitor	814 (0.20)	733 (1.47)	26 (1.46)
Any systemic therapy	12,920 (3.16)	8693 (17.41)	324 (18.19)

**FIGURE 1:**
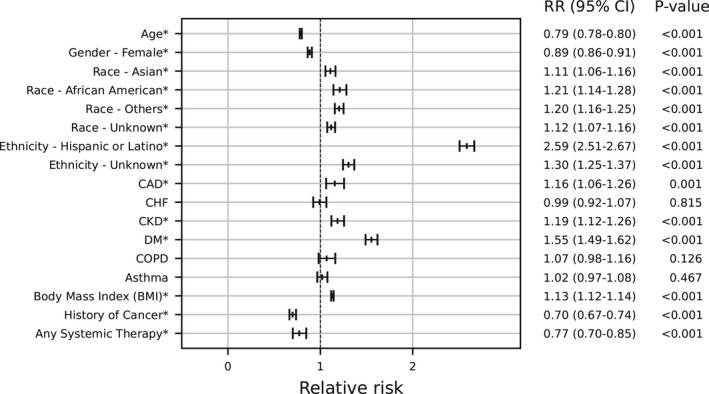
In a multivariable logistic regression of all 409,462 adult patients who underwent SARS‐CoV‐2 testing, race, ethnicity, and comorbidities were associated with an increased risk of a positive test. History of cancer and antineoplastic systemic therapy were associated with a decreased risk of a positive test. Adjusted relative risk ratios are shown. CAD, coronary artery disease; CHF, congestive heart failure; COPD, chronic obstructive pulmonary disease. * Denotes *p* < 0.05

Figure 1 describes factors associated with SARS‐CoV‐2 test positivity in the entire cohort. In terms of cancer‐related factors, positive cancer history was associated with a decreased risk of a positive test (RR 0.70, 95% CI: 0.67–0.74, *p* < 0.001). Similarly, any systemic therapy use was associated with a decreased risk of a positive test (RR 0.77, 95% CI: 0.70–0.85, *p* < 0.001). In terms of specific cancer types, many were associated with a decreased risk or no difference in risk of a positive test compared to unspecified cancer types (Figure S2).

**FIGURE 2:**
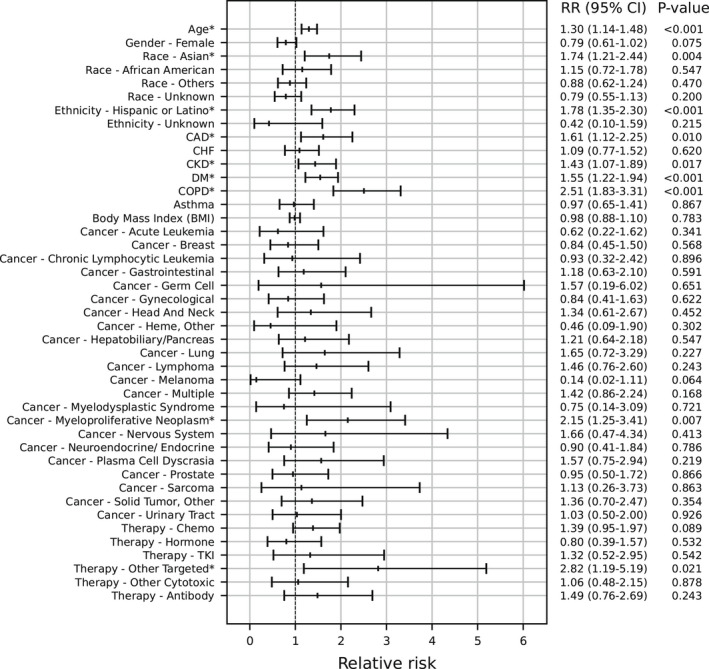
Adjusted risk of 30‐day hospitalization following a positive SARS‐CoV‐2 test among 1781 adult cancer patients. In a multivariable logistic regression of 1781 adult patients with a history of cancer and a positive SARS‐CoV‐2 test, Asian race (compared to White race), Hispanic/Latino ethnicity (compared to not Hispanic/Latino ethnicity), multiple comorbidities, myeloproliferative neoplasm (compared to unspecified cancer type), and Other targeted therapy (compared to not) were associated with an increased risk of 30‐day hospitalization. Adjusted relative risk ratios are shown. CAD, coronary artery disease; CHF, congestive heart failure; COPD, chronic obstructive pulmonary disease; TKI, tyrosine kinase inhibitor. Chronic Myelogenous Leukemia (N = 11) was combined with Cancer‐Hematologic, other given few patients. * Denotes *p* < 0.05

**FIGURE 3:**
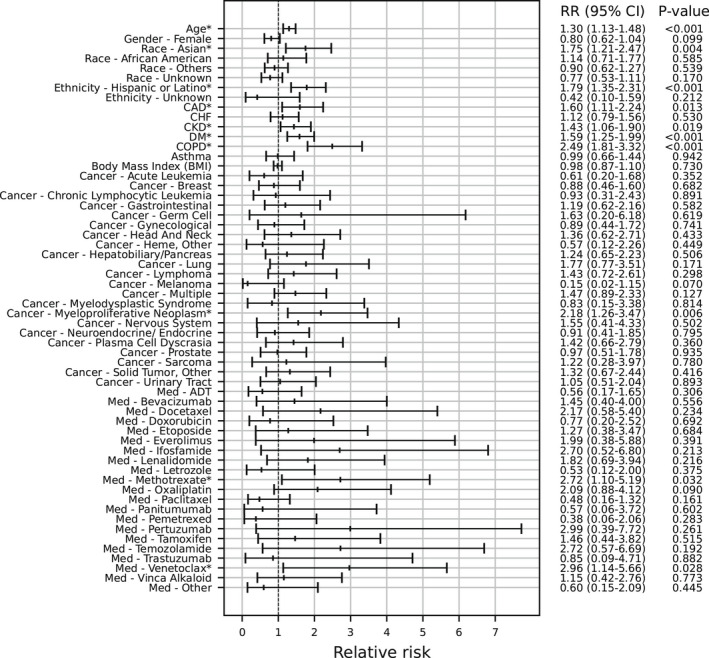
This multivariable logistic regression is identical to that in Figure 2, except that individual systemic therapies are delineated. Methotrexate (categorized as chemotherapy) and venetoclax (categorized as Other targeted therapy) were associated with an increased risk of 30‐day hospitalization. Adjusted relative risk ratios are shown. ADT, androgen deprivation therapy; CAD, coronary artery disease; CHF, congestive heart failure; COPD, chronic obstructive pulmonary disease. Exemestane (N = 11) was combined with Med‐Letrozole given few patients. Med‐Other includes Dasatinib (N=7), Ruxolitinib (N = 7), and Sirolimus (N = 8). * Denotes *p* < 0.05

Among 1781 patients with cancer, 388 (21.8%) were hospitalized within 30 days after the index date. In the multivariable analysis, risk factors for hospitalization included older age, Asian race (compared to White), Hispanic/Latino ethnicity, and several comorbidities (i.e., coronary artery disease, chronic kidney disease, diabetes mellitus, and chronic obstructive pulmonary disease; Figure 2). In terms of cancer type, only myeloproliferative neoplasm (which includes polycythemia vera, essential thrombocythemia, and primary myelofibrosis and does not include chronic myeloid leukemia) was associated with an increased risk of hospitalization compared to unspecified cancer type (RR 2.15, 95% CI: 1.25–3.41, *p* = 0.007; Figure 2). For antineoplastic systemic therapies, chemotherapy was not associated with an increased risk of hospitalization (RR 1.39, 95% CI: 0.95–1.97, *p* = 0.089) and only other targeted therapy was associated with an increased risk (RR 2.82, 95% CI: 1.19–5.19, *p* = 0.021). In the model with individual therapies, methotrexate (RR 2.72, 95% CI: 1.10–5.19, *p* = 0.032) and venetoclax (RR 2.96, 95% CI: 1.14–5.66, *p* = 0.028) were associated with an increased risk of hospitalization (Figure 3). Of note, venetoclax was categorized as other targeted therapy.

In a post hoc sensitivity analysis of this model, we changed the time window of therapy receipt from 60 days before through 30 days after positive test date to 60 days before the positive test date. Methotrexate remained associated with an increased risk of hospitalization (RR 2.98, 95% CI: 1.06–5.86, *p* = 0.040), as did venetoclax (RR 2.97, 95% CI: 1.04–5.87, *p* = 0.042; data not shown).

**FIGURE 4:**
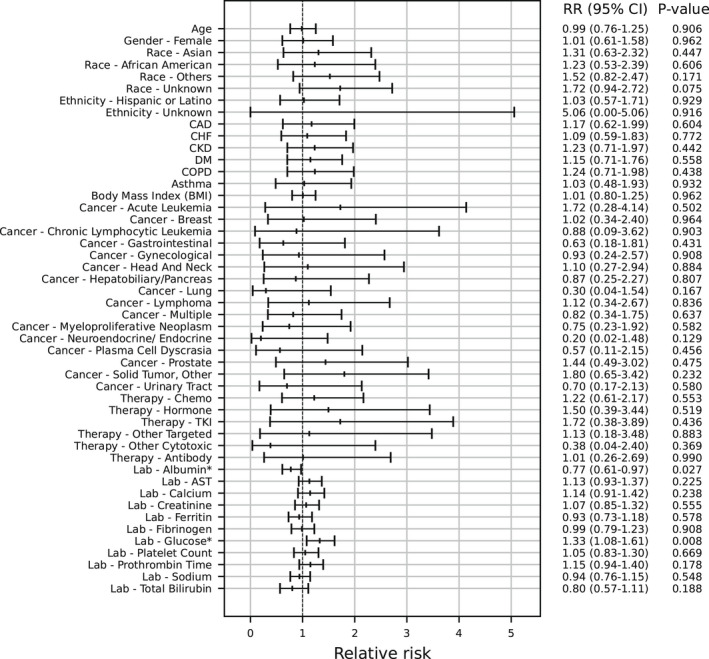
In a multivariable logistic regression of 388 adult patients with a history of cancer who were hospitalized within 30 days after a positive SARS‐CoV‐2 test, no factors were associated with an increased risk of severe COVID‐19. Severe COVID‐19 was defined as intensive care admission, receipt of mechanical ventilation, or death within 30 days of first positive test. Adjusted relative risk ratios are shown. CAD, coronary artery disease; CHF, congestive heart failure. COPD, chronic obstructive pulmonary disease. TKI, tyrosine kinase inhibitor. * Denotes *p* < 0.05

Among 388 cancer patients who were hospitalized after a positive SARS‐CoV‐2 test, 160 (41.2%) had severe COVID‐19. In multivariable analysis, no demographic, comorbidity, or cancer‐related factors were associated with a decrease in risk of severe COVID‐19 (Figure 4). Higher albumin was associated with a decreased risk (RR 0.77, 95% CI: 0.61–0.97, *p* = 0.027), and higher glucose was associated with an increased risk (RR 1.33, 95% CI: 1.08–1.61, *p* = 0.008) of severe COVID‐19. Myeloproliferative neoplasm (N = 32) was not associated with severe COVID‐19 (RR 0.75, 95% CI: 0.23–1.92, *p* = 0.582) compared to unspecified cancer type.

With the hypothesis that COVID‐19 severity for patients with myeloproliferative neoplasms varies based on abnormalities in thrombosis‐related laboratory values, we conducted a *post hoc* analysis in which we added interaction terms between myeloproliferative neoplasms and platelet count and between myeloproliferative neoplasms and fibrinogen to the model. The interaction terms were not significant (Figure S3).

## 4 DISCUSSION

In this study, we used a state‐wide multi‐institution collection of electronic health record data comprising all patients undergoing SARS‐CoV‐2 testing in UC health systems. We identified cancer‐related factors associated with adverse outcomes that had not previously been described.

Notably, we found a decreased risk of SARS‐CoV‐19 positivity in patients with cancer compared to those without cancer. This is counter to studies that report an increased risk of infection in patients with cancer,^2^, ^7^, ^18^ and others that report no difference in risk.^19^, ^20^ The discrepancy between these studies and ours may be related to greater protective behaviors and testing practices in UC patients with cancer. For example, UC patients with cancer may be likely to employ behaviors that decrease transmission (e.g., social distancing and mask‐wearing) or carry a lower threshold to undergo testing compared to patients with cancer in other regions. There is also likely selection bias, as UC patients with cancer may have undergone more tests than those without cancer due to mandated asymptomatic testing prior to infusions, radiation therapy, and surgeries, as had been instituted in some UC medical centers. A similar selection bias was found to potentially explain the association between allergy medication use and decreased SARS‐CoV‐2 infection.^21^ We found that patients receiving systemic therapy were also less likely to test positive, perhaps for similar reasons. Therefore, these findings should be interpreted with caution. Future studies could examine patient behaviors and SARS‐CoV‐2 testing indication to better investigate this discrepancy.

With more granular investigation of the role of cancer types and therapies, we also identified previously undescribed risk factors for hospitalization in patients with cancer. Patients with BCR/ABL‐negative myeloproliferative neoplasms and COVID‐19 were at an increased risk of hospitalization. Patients with myeloproliferative neoplasms are in a pro‐inflammatory state, with qualitative and quantitative abnormalities in myeloid cells leading to both venous/arterial thrombosis and coagulopathy.^22^, ^23^, ^24^ Similarly, COVID‐19 severity is closely related to pro‐inflammatory markers,^25^ and is also associated with both thrombosis and coagulopathy.^26^, ^27^ Therefore, patients with myeloproliferative neoplasms may be particularly susceptible to worse COVID‐19 outcomes.^28^ To our knowledge, this finding has not been previously described in comparative studies, perhaps because these patients have been excluded or under‐represented in cancer cohort studies. Some supportive evidence does exist. In a non‐comparative study, Salisbury et al.^29^ highlighted a high rate of adverse outcomes in patients with myeloproliferative neoplasms and COVID‐19, especially upon ruxolitinib withdrawal. Other groups have reported a similar or decreased risk of mortality in patients with myeloproliferative neoplasms compared to those with other hematologic malignancies.^30^, ^31^, ^32^ In our study, we did not find that hospitalized patients with myeloproliferative neoplasms had a higher risk of severe COVID‐19, but this analysis is limited by the small size of this subgroup.

Two antineoplastic medications were found to be associated with an increased risk of hospitalization. Venetoclax, a Bcl2 inhibitor, is commonly used in the treatment of chronic lymphocytic leukemia as a monotherapy, and in combination with other therapies for acute myeloid leukemia. Adverse outcomes have been previously described in patients receiving venetoclax for chronic lymphocytic leukemia in a non‐comparative study, but not in any large, comparative study to our knowledge.^33^ Its association with an increased risk of hospitalization may be related to the negative effect on immune function via lymphocyte depletion, a known risk factor for COVID‐19 severity,^34^ and via reduced interferon‐alpha production and dendritic cells depletion; pneumonia is a known toxicity or venetoclax.^35^ Another potential mechanism involves ACE‐2 and bcl‐2. Motaghinejad et al.^36^ postulated that increased COVID‐19 mortality is partially driven by decreased ACE2 expression in the pulmonary and cardiovascular systems, causing destabilization of Bcl‐2 and dysregulation of apoptosis. This dysregulation may be compounded for patients receiving venetoclax, a Bcl‐2 inhibitor, leading to cardiopulmonary complications.

Methotrexate was also associated with an increased risk of hospitalization. As cytotoxic chemotherapy, methotrexate may increase susceptibility to COVID‐19 complications through immunosuppression. Though most studies suggest that chemotherapy, in general, is a risk factor for worse COVID‐19 outcomes, several studies have not confirmed the association, likely due to the heterogeneity of different chemotherapy agents and regimens.^4^, ^9^, ^37^ Methotrexate as a risk factor has not been studied in cancer patients, but findings for low‐dose methotrexate in patients with rheumatologic conditions have been mixed.^38^, ^39^ Despite the biologic rationale for poor COVID‐19 outcomes in patients receiving venetoclax or methotrexate, we did not find a consistent association across other outcomes. For example, there was no increased risk of severe COVID‐19 in hospitalized patients receiving these therapies. Though this negative finding should be interpreted with caution given the small sample sizes, we cannot exclude the possibility that these observations are coincidental given the high number of individual therapies included in the models. Further confirmatory studies investigating these potential risk factors should be performed.

We also identified known risk factors for hospitalization following COVID‐19. These risk factors included older age, Asian race, Hispanic or Latino ethnicity, coronary artery disease, chronic kidney disease, diabetes mellitus, and chronic obstructive pulmonary disease. Prior studies have demonstrated these associations.^5^, ^8^, ^40^, ^41^, ^42^

In our study, we also demonstrated the feasibility and utility of a prospectively created, frequently, and passively updated multi‐center dataset. In the COVID‐19 and future pandemics, it is critical to rapidly identify patient‐related attributes and interventions that affect the risk of infection, morbidity, and mortality. The timely creation of frequently and passively updated data sources that contain patient‐level clinical data from multiple health systems, like UC CORDS and N3C, is invaluable to this goal. These data could complement those of other consortium and registry efforts, such as the CCC19, which provide more granular data using human abstraction.^4^ For example, Reznikov et al.^18^ mined UC CORDS within a few months of its creation and identified antihistamines associated with decreased SARS‐CoV‐2 test positivity. In vitro drug susceptibility assays showed hypothesis‐generating antiviral mechanisms of candidate antihistamines.

Central data sets for future pandemics could include other data types (e.g., imaging, patient symptoms, and genomic data to identify mechanisms and therapeutic targets); real‐time data collection (e.g., patient location, activity, and vital data using wearable devices); and data from other health care systems (e.g., Veterans Health Administration and Community Health Centers). Pandemic preparation should also include identification of and resource‐allocation to teams to harmonize and standardize raw data, and to query, analyze, and interpret databases. Efforts must be done ethically with potential biases in mind.^43^ Moreover, advanced analytic techniques, such as artificial intelligence (AI) approaches, that are findable, accessible, interoperable, and reusable to facilitate the development of new AI applications, could be applied.

This study has several strengths, including one of the largest cancer cohorts to date, a diverse cohort, use of a novel database, and more granular categorization of cancer types and therapies. There are several limitations. Selection bias may have been introduced from the inclusion of only patients who underwent SARS‐CoV‐2 testing and from over‐representation of patients cared for at academic centers, where asymptomatic testing and COVID‐19 treatment practices may have differed from other hospitals. The database does not contain certain risk factors for severe COVID‐19, including cancer stage, cancer remission status, smoking status, poor performance status, and socioeconomic variables. Due to the limited sample size of hospitalized patients, we could not evaluate whether venetoclax and methotrexate were associated with severe COVID‐19 infection. Multiple testing was not accounted for. Lastly, we could not ascertain the outcomes of patients who sought care outside the UC health system.

## 5 CONCLUSION

As the COVID‐19 pandemic continues and new, highly transmissible variants emerge, it is important to remain vigilant of risk factors for severe infection. Close attention to patients with risk factors will allow us to better prevent and monitor COVID‐19 in high‐risk patients. We found that patients with COVID‐19 and myeloproliferative neoplasms, and those receiving methotrexate or venetoclax, may be at an increased risk of hospitalization. Further studies to confirm these associations are needed, as are studies to understand underlying mechanisms. Investigation is also needed to explain and confirm the lower risk of test positivity in patients with cancer than those without cancer. Lastly, policy makers and health systems should focus on establishing timely, live central databases of electronic health data to provide rapidly accumulating data for future pandemic preparedness, as well as the human capital needed for their maintenance and use.

## CONFLICT OF INTEREST

None.

## ETHICAL APPROVAL STATEMENT

All procedures performed in studies involving human participants were in accordance with the ethical standards of UCSF and LLNL research committees and with the 1964 Helsinki declaration and its later amendments or comparable ethical standards.

## AUTHOR CONTRIBUTIONS

*Conceptualization*: Daniel H. Kwon, Amy L. Gryshuk, Priyadip Ray, Franklin W. Huang *Data curation*: Daniel H. Kwon, Franklin W. Huang *Formal analysis*: Daniel H. Kwon, Jose Cadena, Sam Nguyen, Kwan Ho Ryan Chan, Braden Soper, Priyadip Ray, Franklin W. Huang *Funding acquisition*: Priyadip Ray *Investigation*: Daniel H. Kwon, Jose Cadena, Sam Nguyen, Kwan Ho Ryan Chan, Braden Soper, Priyadip Ray *Methodology*: Daniel H. Kwon, Priyadip Ray, Franklin W. Huang, J.C.H. *Project administration*: Daniel H. Kwon, Priyadip Ray, Franklin W. Huang *Resources*: Daniel H. Kwon, Priyadip Ray, Franklin W. Huang *Software*: Sam Nguyen, Kwan Ho Ryan Chan, Braden Soper, Priyadip Ray *Supervision*: Franklin W. Huang *Validation*: n/a. *Visualization*: Daniel H. Kwon, Jose Cadena *Writing – original draft*: Daniel H. Kwon *Writing – review & editing*: all authors.

## Supporting information


FigureS1

FigureS2

FigureS3

TableS1‐S3

## Data Availability

The data that support the findings of this study are available from the corresponding author upon reasonable request.
