# Supplementary material for: COVID‐19 outcomes in patients with cancer: Findings from the University of California health system database
Source: Cancer Med. 2022 Mar 9;11(11):2204–15. doi: 10.1002/cam4.4604 (PMC9110901; doi:10.1002/cam4.4604)
Supplement: Supplementary file 1 — FigureS1 [file CAM4-11-2204-s002.pdf]

Supplementary Figure S1: Flow diagram illustrating study cohort selection for analyses

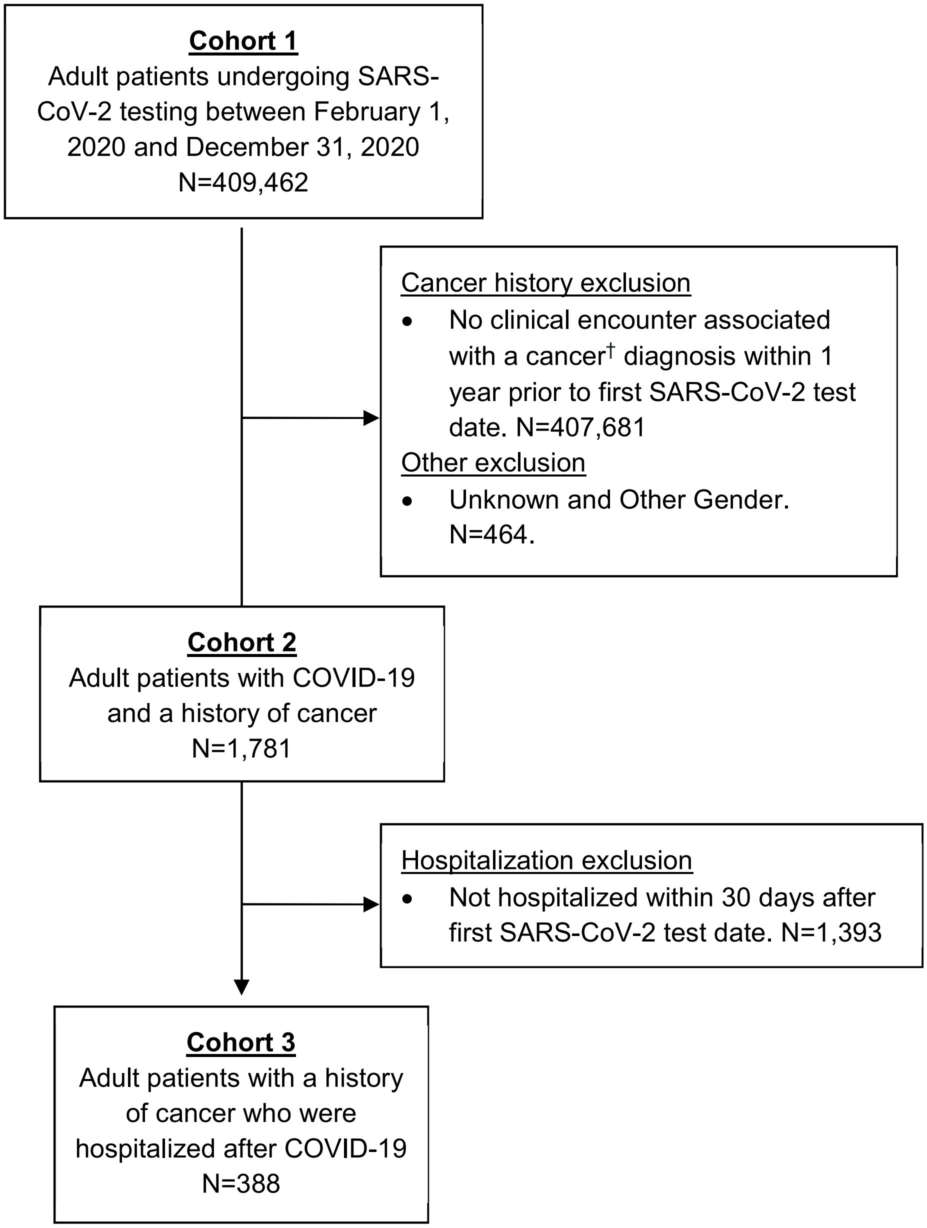

† Patients with basal and squamous cell cutaneous cancers were excluded.
