# Supplementary material for: COVID‐19 outcomes in patients with cancer: Findings from the University of California health system database
Source: Cancer Med. 2022 Mar 9;11(11):2204–15. doi: 10.1002/cam4.4604 (PMC9110901; doi:10.1002/cam4.4604)
Supplement: Supplementary file 4 — TableS1‐S3 [file CAM4-11-2204-s003.docx]

**Supplementary Table S1. ICD10CM codes used for “Unspecified cancer type”**

| **Diagnosis** | **Code** |
| --- | --- |
| Aftercare following surgery for neoplasm | Z48.3 |
| Agranulocytosis secondary to cancer chemotherapy | D70.1 |
| Disorders of optic chiasm in (due to) neoplasm | H47.42 |
| Disorders of visual cortex in (due to) neoplasm | H47.63 |
| Disorders of visual cortex in (due to) neoplasm, left side of brain | H47.632 |
| Disorders of visual cortex in (due to) neoplasm, right side of brain | H47.631 |
| Disorders of visual cortex in (due to) neoplasm, unspecified side of brain | H47.639 |
| Disorders of visual pathways in (due to) neoplasm | H47.52 |
| Disorders of visual pathways in (due to) neoplasm, left side | H47.522 |
| Disorders of visual pathways in (due to) neoplasm, right side | H47.521 |
| Disorders of visual pathways in (due to) neoplasm, unspecified side | H47.529 |
| Disseminated malignant neoplasm, unspecified | C80.0 |
| Malignant (primary) neoplasm, unspecified | C80.1 |
| Malignant ascites | R18.0 |
| Malignant neoplasm associated with transplanted organ | C80.2 |
| Malignant neoplasm complicating childbirth | O9A.12 |
| Malignant neoplasm complicating pregnancy | O9A.11 |
| Malignant neoplasm complicating pregnancy, childbirth and the puerperium | O9A.1 |
| Malignant neoplasm complicating pregnancy, first trimester | O9A.111 |
| Malignant neoplasm complicating pregnancy, second trimester | O9A.112 |
| Malignant neoplasm complicating pregnancy, third trimester | O9A.113 |
| Malignant neoplasm complicating pregnancy, unspecified trimester | O9A.119 |
| Malignant neoplasm complicating the puerperium | O9A.13 |
| Malignant neoplasm of abdomen | C76.2 |
| Malignant neoplasm of anterior mediastinum | C38.1 |
| Malignant neoplasm of bone and articular cartilage of limbs | C40 |
| Malignant neoplasm of bone and articular cartilage of other and unspecified sites | C41 |
| Malignant neoplasm of brain | C71 |
| Malignant neoplasm of brain stem | C71.7 |
| Malignant neoplasm of brain, unspecified | C71.9 |
| Malignant neoplasm of carotid body | C75.4 |
| Malignant neoplasm of cauda equina | C72.1 |
| Malignant neoplasm of central nervous system, unspecified | C72.9 |
| Malignant neoplasm of cerebellum | C71.6 |
| Malignant neoplasm of cerebral meninges | C70.0 |
| Malignant neoplasm of cerebral ventricle | C71.5 |
| Malignant neoplasm of cerebrum, except lobes and ventricles | C71.0 |
| Malignant neoplasm of frontal lobe | C71.1 |
| Malignant neoplasm of left acoustic nerve | C72.42 |
| Malignant neoplasm of left lower limb | C76.52 |
| Malignant neoplasm of left olfactory nerve | C72.22 |
| Malignant neoplasm of left optic nerve | C72.32 |
| Malignant neoplasm of left upper limb | C76.42 |
| Malignant neoplasm of long bones of left lower limb | C40.22 |
| Malignant neoplasm of long bones of lower limb | C40.2 |
| Malignant neoplasm of long bones of right lower limb | C40.21 |
| Malignant neoplasm of long bones of unspecified lower limb | C40.20 |
| Malignant neoplasm of lower limb | C76.5 |
| Malignant neoplasm of meninges | C70 |
| Malignant neoplasm of meninges, unspecified | C70.9 |
| Malignant neoplasm of occipital lobe | C71.4 |
| Malignant neoplasm of olfactory nerve | C72.2 |
| Malignant neoplasm of optic nerve | C72.3 |
| Malignant neoplasm of other and ill-defined sites | C76 |
| Malignant neoplasm of other and ill-defined sites in the respiratory system and intrathoracic organs | C39 |
| Malignant neoplasm of other and unspecified cranial nerves | C72.5 |
| Malignant neoplasm of other and unspecified male genital organs | C63 |
| Malignant neoplasm of other cranial nerves | C72.59 |
| Malignant neoplasm of other ill-defined sites | C76.7 |
| Malignant neoplasm of other specified ill-defined sites | C76.8 |
| Malignant neoplasm of overlapping sites of bone and articular cartilage of left limb | C40.82 |
| Malignant neoplasm of overlapping sites of bone and articular cartilage of limb | C40.8 |
| Malignant neoplasm of overlapping sites of bone and articular cartilage of right limb | C40.81 |
| Malignant neoplasm of overlapping sites of bone and articular cartilage of unspecified limb | C40.80 |
| Malignant neoplasm of overlapping sites of brain | C71.8 |
| Malignant neoplasm of overlapping sites of connective and soft tissue | C49.8 |
| Malignant neoplasm of overlapping sites of eye and adnexa | C69.8 |
| Malignant neoplasm of overlapping sites of left eye and adnexa | C69.82 |
| Malignant neoplasm of overlapping sites of male genital organs | C63.8 |
| Malignant neoplasm of overlapping sites of peripheral nerves and autonomic nervous system | C47.8 |
| Malignant neoplasm of overlapping sites of right eye and adnexa | C69.81 |
| Malignant neoplasm of overlapping sites of unspecified eye and adnexa | C69.80 |
| Malignant neoplasm of parietal lobe | C71.3 |
| Malignant neoplasm of pelvis | C76.3 |
| Malignant neoplasm of peripheral nerves and autonomic nervous system | C47 |
| Malignant neoplasm of peripheral nerves and autonomic nervous system, unspecified | C47.9 |
| Malignant neoplasm of peripheral nerves of abdomen | C47.4 |
| Malignant neoplasm of peripheral nerves of head, face and neck | C47.0 |
| Malignant neoplasm of peripheral nerves of left lower limb, including hip | C47.22 |
| Malignant neoplasm of peripheral nerves of left upper limb, including shoulder | C47.12 |
| Malignant neoplasm of peripheral nerves of lower limb, including hip | C47.2 |
| Malignant neoplasm of peripheral nerves of pelvis | C47.5 |
| Malignant neoplasm of peripheral nerves of right lower limb, including hip | C47.21 |
| Malignant neoplasm of peripheral nerves of right upper limb, including shoulder | C47.11 |
| Malignant neoplasm of peripheral nerves of thorax | C47.3 |
| Malignant neoplasm of peripheral nerves of trunk, unspecified | C47.6 |
| Malignant neoplasm of peripheral nerves of unspecified lower limb, including hip | C47.20 |
| Malignant neoplasm of peripheral nerves of unspecified upper limb, including shoulder | C47.10 |
| Malignant neoplasm of peripheral nerves of upper limb, including shoulder | C47.1 |
| Malignant neoplasm of pineal gland | C75.3 |
| Malignant neoplasm of placenta | C58 |
| Malignant neoplasm of posterior mediastinum | C38.2 |
| Malignant neoplasm of ribs, sternum and clavicle | C41.3 |
| Malignant neoplasm of right acoustic nerve | C72.41 |
| Malignant neoplasm of right lower limb | C76.51 |
| Malignant neoplasm of right olfactory nerve | C72.21 |
| Malignant neoplasm of right optic nerve | C72.31 |
| Malignant neoplasm of right upper limb | C76.41 |
| Malignant neoplasm of scapula and long bones of upper limb | C40.0 |
| Malignant neoplasm of short bones of left lower limb | C40.32 |
| Malignant neoplasm of short bones of left upper limb | C40.12 |
| Malignant neoplasm of short bones of lower limb | C40.3 |
| Malignant neoplasm of short bones of right lower limb | C40.31 |
| Malignant neoplasm of short bones of right upper limb | C40.11 |
| Malignant neoplasm of short bones of unspecified lower limb | C40.30 |
| Malignant neoplasm of short bones of unspecified upper limb | C40.10 |
| Malignant neoplasm of short bones of upper limb | C40.1 |
| Malignant neoplasm of spinal cord | C72.0 |
| Malignant neoplasm of spinal cord, cranial nerves and other parts of central nervous system | C72 |
| Malignant neoplasm of spinal meninges | C70.1 |
| Malignant neoplasm of spleen | C26.1 |
| Malignant neoplasm of temporal lobe | C71.2 |
| Malignant neoplasm of thorax | C76.1 |
| Malignant neoplasm of unspecified acoustic nerve | C72.40 |
| Malignant neoplasm of unspecified bones and articular cartilage of left limb | C40.92 |
| Malignant neoplasm of unspecified bones and articular cartilage of limb | C40.9 |
| Malignant neoplasm of unspecified bones and articular cartilage of right limb | C40.91 |
| Malignant neoplasm of unspecified bones and articular cartilage of unspecified limb | C40.90 |
| Malignant neoplasm of unspecified cranial nerve | C72.50 |
| Malignant neoplasm of unspecified lower limb | C76.50 |
| Malignant neoplasm of unspecified olfactory nerve | C72.20 |
| Malignant neoplasm of unspecified optic nerve | C72.30 |
| Malignant neoplasm of unspecified upper limb | C76.40 |
| Malignant neoplasm of upper limb | C76.4 |
| Maternal malignant neoplasms, traumatic injuries and abuse classifiable elsewhere but complicating pregnancy, childbirth and the puerperium | O9A |
| Neoplasm of uncertain behavior of brain and central nervous system | D43 |
| Neoplasm of uncertain behavior of central nervous system, unspecified | D43.9 |
| Neoplasm of uncertain behavior of cerebral meninges | D42.0 |
| Neoplasm of uncertain behavior of connective and other soft tissue | D48.1 |
| Neoplasm of uncertain behavior of cranial nerves | D43.3 |
| Neoplasm of uncertain behavior of meninges | D42 |
| Neoplasm of uncertain behavior of meninges, unspecified | D42.9 |
| Neoplasm of uncertain behavior of other and unspecified sites | D48 |
| Neoplasm of uncertain behavior of other parts of central nervous system | D43.7 |
| Neoplasm of uncertain behavior of other specified parts of central nervous system | D43.8 |
| Neoplasm of uncertain behavior of peripheral nerves and autonomic nervous system | D48.2 |
| Neoplasm of uncertain behavior of peritoneum | D48.4 |
| Neoplasm of uncertain behavior of placenta | D39.2 |
| Neoplasm of uncertain behavior of retroperitoneum | D48.3 |
| Neoplasm of uncertain behavior of spinal meninges | D42.1 |
| Neoplasm of unspecified behavior of unspecified site | D49.9 |
| Neoplasms of unspecified behavior | D49 |
| Neoplastic (malignant) related fatigue | R53.0 |
| Secondary and unspecified malignant neoplasm of axilla and upper limb lymph nodes | C77.3 |
| Secondary and unspecified malignant neoplasm of inguinal and lower limb lymph nodes | C77.4 |
| Secondary and unspecified malignant neoplasm of intra-abdominal lymph nodes | C77.2 |
| Secondary and unspecified malignant neoplasm of intrapelvic lymph nodes | C77.5 |
| Secondary and unspecified malignant neoplasm of intrathoracic lymph nodes | C77.1 |
| Secondary and unspecified malignant neoplasm of lymph node, unspecified | C77.9 |
| Secondary and unspecified malignant neoplasm of lymph nodes | C77 |
| Secondary and unspecified malignant neoplasm of lymph nodes of head, face and neck | C77.0 |
| Secondary and unspecified malignant neoplasm of lymph nodes of multiple regions | C77.8 |
| Secondary malignant neoplasm of adrenal gland | C79.7 |
| Secondary malignant neoplasm of bladder | C79.11 |
| Secondary malignant neoplasm of bladder and other and unspecified urinary organs | C79.1 |
| Secondary malignant neoplasm of bone | C79.51 |
| Secondary malignant neoplasm of bone and bone marrow | C79.5 |
| Secondary malignant neoplasm of bone marrow | C79.52 |
| Secondary malignant neoplasm of brain | C79.31 |
| Secondary malignant neoplasm of brain and cerebral meninges | C79.3 |
| Secondary malignant neoplasm of breast | C79.81 |
| Secondary malignant neoplasm of cerebral meninges | C79.32 |
| Secondary malignant neoplasm of genital organs | C79.82 |
| Secondary malignant neoplasm of kidney and renal pelvis | C79.0 |
| Secondary malignant neoplasm of large intestine and rectum | C78.5 |
| Secondary malignant neoplasm of left adrenal gland | C79.72 |
| Secondary malignant neoplasm of left kidney and renal pelvis | C79.02 |
| Secondary malignant neoplasm of left lung | C78.02 |
| Secondary malignant neoplasm of left ovary | C79.62 |
| Secondary malignant neoplasm of liver and intrahepatic bile duct | C78.7 |
| Secondary malignant neoplasm of lung | C78.0 |
| Secondary malignant neoplasm of mediastinum | C78.1 |
| Secondary malignant neoplasm of other and unspecified digestive organs | C78.8 |
| Secondary malignant neoplasm of other and unspecified parts of nervous system | C79.4 |
| Secondary malignant neoplasm of other and unspecified respiratory organs | C78.3 |
| Secondary malignant neoplasm of other and unspecified sites | C79 |
| Secondary malignant neoplasm of other digestive organs | C78.89 |
| Secondary malignant neoplasm of other parts of nervous system | C79.49 |
| Secondary malignant neoplasm of other respiratory organs | C78.39 |
| Secondary malignant neoplasm of other specified sites | C79.8 |
| Secondary malignant neoplasm of other specified sites | C79.89 |
| Secondary malignant neoplasm of other urinary organs | C79.19 |
| Secondary malignant neoplasm of ovary | C79.6 |
| Secondary malignant neoplasm of pleura | C78.2 |
| Secondary malignant neoplasm of respiratory and digestive organs | C78 |
| Secondary malignant neoplasm of retroperitoneum and peritoneum | C78.6 |
| Secondary malignant neoplasm of right adrenal gland | C79.71 |
| Secondary malignant neoplasm of right kidney and renal pelvis | C79.01 |
| Secondary malignant neoplasm of right lung | C78.01 |
| Secondary malignant neoplasm of right ovary | C79.61 |
| Secondary malignant neoplasm of skin | C79.2 |
| Secondary malignant neoplasm of small intestine | C78.4 |
| Secondary malignant neoplasm of unspecified adrenal gland | C79.70 |
| Secondary malignant neoplasm of unspecified digestive organ | C78.80 |
| Secondary malignant neoplasm of unspecified kidney and renal pelvis | C79.00 |
| Secondary malignant neoplasm of unspecified lung | C78.00 |
| Secondary malignant neoplasm of unspecified ovary | C79.60 |
| Secondary malignant neoplasm of unspecified part of nervous system | C79.40 |
| Secondary malignant neoplasm of unspecified respiratory organ | C78.30 |
| Secondary malignant neoplasm of unspecified site | C79.9 |
| Secondary malignant neoplasm of unspecified urinary organs | C79.10 |

**Supplementary Table S2. Categorization of antineoplastic systemic therapies**

|  | **Antibody** | **Chemo-therapy** | **Hormone therapy** | **Immune-based therapy** | **Tyrosine kinase inhibitor** | **Other cytotoxic therapy** | **Other targeted therapy** |
| --- | --- | --- | --- | --- | --- | --- | --- |
| Anti-EGFR: e.g., cetuximab | x |  |  |  |  |  |  |
| Anti-HER2: e.g., trastuzumab | x |  |  |  |  |  |  |
| Anti-CD20: e.g., rituximab | x |  |  |  |  |  |  |
| Anti-CD38: e.g., daratumumab | x |  |  |  |  |  |  |
| Anti-angiogenesis: e.g., bevacizumab | x |  |  |  |  |  |  |
| Other: e.g., elotuzumab | x |  |  |  |  |  |  |
| Cytotoxic chemotherapy: e.g., methotrexate |  | x |  |  |  |  |  |
| Androgen deprivation therapies: e.g., leuprolide, degarelix, abiraterone |  |  | x |  |  |  |  |
| Estrogen-targeted therapies: e.g., tamoxifen |  |  | x |  |  |  |  |
| Checkpoint inhibitors: e.g., pembrolizumab |  |  |  | x |  |  |  |
| Cellular therapies: e.g., tisagenlecleucel |  |  |  | x |  |  |  |
| Cancer vaccines: e.g., sipuleucel-T |  |  |  | x |  |  |  |
| Immunostimulants: e.g., interferon-alfa 2b |  |  |  | x |  |  |  |
| VEGF inhibitor: e.g., sunitinib |  |  |  |  | x |  |  |
| BCR/ABL inhibitor: e.g., imatinib |  |  |  |  | x |  |  |
| JAK2 inhibitor: e.g., ruxolitinib |  |  |  |  | x |  |  |
| BTK inhibitor: e.g., ibrutinib |  |  |  |  | x |  |  |
| EGFR inhibitor: e.g., erlotinib |  |  |  |  | x |  |  |
| Other: e.g., vandetanib |  |  |  |  | x |  |  |
| mTOR inhibitor: e.g., everolimus |  |  |  |  |  | x |  |
| CDK4/6 inhibitor: e.g., palbociclib |  |  |  |  |  | x |  |
| Other: e.g., lenalidomide |  |  |  |  |  | x |  |
| HDAC inhibitor: e.g., belinostat |  |  |  |  |  | x |  |
| BCL2 inhibitor: e.g., venetoclax |  |  |  |  |  |  | x |
| PARP inhibitor: e.g., olaparib |  |  |  |  |  |  | x |
| Antibody-drug conjugate: e.g., enfortumab vedotin |  |  |  |  |  |  | x |
| PI3K inhibitor: e.g., alpelisib |  |  |  |  |  |  | x |
| BRAF/MEK inhibitor: e.g., dabrafenib |  |  |  |  |  |  | x |
| Other: e.g., enasidenib |  |  |  |  |  |  | x |

**Supplementary Table S3. Antineoplastic systemic therapies received by 1,781 cancer patients undergoing SARS-CoV-2 testing**

| **Therapy** | **Category** | **N (%)** |
| --- | --- | --- |
| Androgen deprivation therapies (e.g.,  leuprolide acetate, degarelix, abiraterone etc.) | Hormone | 42 (2.31) |
| Arsenic | Chemotherapy | 1 (0.06) |
| Bevacizumab | Antibody | 13 (0.72) |
| Blinatumomab | Antibody | 2 (0.11) |
| Cladribine | Chemotherapy | 1 (0.06) |
| Clofarabine | Chemotherapy | 1 (0.06) |
| Cyclophosphamide | Chemotherapy | 2 (0.11) |
| Cytarabine | Chemotherapy | 1 (0.06) |
| Daratumumab | Antibody | 4 (0.22) |
| Dasatinib | TKI | 7 (0.39) |
| Decitabine | Chemotherapy | 1 (0.06) |
| Docetaxel | Chemotherapy | 9 (0.50) |
| Doxorubicin | Chemotherapy | 20 (1.10) |
| Encorafenib | Other targeted | 2 (0.11) |
| Entrectinib | Other targeted | 1 (0.06) |
| Eribulin | Chemotherapy | 1 (0.06) |
| Etoposide | Chemotherapy | 20 (1.10) |
| Everolimus | Other cytotoxic | 6 (0.33) |
| Exemestane | Hormone | 11 (0.61) |
| Fedratinib | TKI | 3 (0.17) |
| Gemcitabine | Chemotherapy | 4 (0.22) |
| Ifosfamide | Chemotherapy | 6 (0.33) |
| Imatinib | TKI | 4 (0.22) |
| Lenalidomide | Other cytotoxic | 20 (1.10) |
| Letrozole | Hormone | 28 (1.54) |
| Lomustine | Chemotherapy | 2 (0.11) |
| Methotrexate | Chemotherapy | 24 (1.32) |
| Mitomycin | Chemotherapy | 1 (0.06) |
| Mitoxantrone | Chemotherapy | 1 (0.06) |
| Neratinib | Other cytotoxic | 1 (0.06) |
| Olaparib | Other targeted | 1 (0.06) |
| Osimertinib | TKI | 3 (0.17) |
| Oxaliplatin | Chemotherapy | 19 (1.05) |
| Paclitaxel | Chemotherapy | 42 (2.31) |
| Palbociclib | Other cytotoxic | 2 (0.11) |
| Panitumumab | Antibody | 6 (0.33) |
| Pemetrexed | Chemotherapy | 8 (0.44) |
| Pertuzumab | Antibody | 10 (0.55) |
| Pomalidomide | Other cytotoxic | 4 (0.22) |
| Ramucirumab | Antibody | 3 (0.17) |
| Regorafenib | TKI | 1 (0.06) |
| Romidepsin | Other cytotoxic | 1 (0.06) |
| Ruxolitinib | TKI | 7 (0.39) |
| Sirolimus | Other cytotoxic | 8 (0.44) |
| Tamoxifen | Hormone | 21 (1.16) |
| Temozolamide | Chemotherapy | 7 (0.39) |
| Thiotepa | Chemotherapy | 1 (0.06) |
| Trastuzumab | Antibody | 11 (0.61) |
| Venetoclax | Other targeted | 14 (0.77) |
| Vinca alkaloid | Chemotherapy | 35 (1.93) |
| Vismodegib | Other targeted | 1 (0.06) |

TKI = Tyrosine Kinase Inhibitor
